# Alternative mRNA Splicing Controls the Functions of the Histone H3K27 Demethylase UTX/KDM6A

**DOI:** 10.3390/cancers15123117

**Published:** 2023-06-08

**Authors:** Omid Fotouhi, Sheikh Nizamuddin, Stephanie Falk, Oliver Schilling, Ruth Knüchel-Clarke, Martin L. Biniossek, H. T. Marc Timmers

**Affiliations:** 1 Department of Urology, Medical Center-University of Freiburg, 79106 Freiburg, Germany; omid.fotouhi@ki.se (O.F.); n.sheikh@dkfz-heidelberg.de (S.N.); 2 German Cancer Consortium (DKTK) Partner Site Freiburg, German Cancer Research Center (DKFZ), 69120 Heidelberg, Germany; oliver.schilling@mol-med.uni-freiburg.de; 3 Max Planck Institute of Immunobiology and Epigenetics, 79108 Freiburg, Germany; falks@ie-freiburg.mpg.de; 4 Institute for Surgical Pathology, Faculty of Medicine, Medical Center-University of Freiburg, University of Freiburg, 79106 Freiburg, Germany; 5 Institute of Pathology, University Hospital RWTH Aachen, 52074 Aachen, Germany; rknuechel-clarke@ukaachen.de; 6 Institute of Molecular Medicine and Cell Research, Faculty of Medicine, University of Freiburg, 79106 Freiburg, Germany; martin.biniossek@mol-med.uni-freiburg.de

**Keywords:** alternative splicing, histone demethylase, histone methylation, cancer biology, chromatin, proteomics, bladder cancer

## Abstract

**Simple Summary:**

UTX/KDM6A is a histone H3K27 demethylase and plays an important role in mammalian development and human diseases such as urothelial cancer. We identified a region encompassing exons 12–17 of UTX that undergoes extensive splicing events. As a result, a nuclear localization sequence located in exon 14 is missing in a considerable portion of UTX transcripts in different cell lines and tissues from normal bladder epithelia and bladder cancer cases. Mass spectrometry analysis showed a role of this region in binding to the epigenetic PR-DUB and MiDAC complexes. UTX was also more extensively bound to chromatin when the alternative splicing region was presented. Our study showed that the alternative splicing of UTX transcripts plays an important role in its functions.

**Abstract:**

The UTX/KDM6A histone H3K27 demethylase plays an important role in development and is frequently mutated in cancers such as urothelial cancer. Despite many studies on UTX proteins, variations in mRNA splicing have been overlooked. Using Nanopore sequencing, we present a comprehensive analysis of UTX/KDM6A splicing events in human cell lines and in tissue samples from bladder cancer cases and normal epithelia. We found that the central region of UTX mRNAs encoded by exons 12 to 17 undergoes extensive alternative splicing. Up to half of all stable mRNAs (8–48% in bladder tissues and 18–58% in cell lines) are represented by the UTX canonical isoform lacking exon 14 encoding a nuclear localization sequence, and hence exon 14-containing UTX isoforms exclusively localize to the nucleus, unlike the cytonuclear localization of the canonical isoform. Chromatin association was also higher for exon-14-containing isoforms compared to the canonical UTX. Using quantitative mass spectrometry, we found that all UTX isoforms integrated into the MLL3 and MLL4, PR-DUB and MiDAC complexes. Interestingly, one of the novel UTX isoforms, which lacks exons 14 and 16, fails to interact with PR-DUB and MiDAC complex members. In conclusion, UTX mRNAs undergo extensive alternative splicing, which controls the subcellular localization of UTX and its interactions with other chromatin regulatory complexes.

## 1. Introduction

Chromatin modifiers play a critical role during normal differentiation and in disease states. The Ubiquitously Transcribed tetratricopeptide repeat (TPR) on chromosome X gene, *UTX* (also often referred to as *KDM6A*), encodes a histone H3K27me3 demethylase. Throughout the remainder of this manuscript, we will refer to *UTX*/*KDM6A* as *UTX* for simplicity. The *UTX* gene is highly mutated in different cancer types [1] and especially in bladder cancer (BLCA), with mutation frequencies of up to 30% [2,3]. Even a normal bladder epithelium contains many discrete regions of clonally expanded cells that harbor independent mutations in *UTX* [4]. Such “morphologically normal” predisposition aberrations were not observed for other frequently mutated genes in BLCA such as *TP53* and *RB1*, which indicates that *UTX* inactivation is an early event in bladder carcinogenesis [5].

The pathophysiological significance of UTX is historically attributed to its C-terminal JmjC domain, which carries the histone demethylation function. However, UTX comprises two other functional regions, an N-terminal TPR region and an intrinsically disordered region (IDR) in the middle of the protein. The TPR is a highly conserved 34–40 amino acid motif tandem repeat, which is often involved in protein–protein interactions [6]. The IDR has been shown to be involved in the formation of biomolecular condensates [7]. UTX is an integral member of the MLL3 and MLL4 histone H3K4 methylation complexes of the COMPASS family of SET1/MLL complexes [8,9,10]. The N-terminal PHD region of MLL3/KMT2C has been shown to bind the BAP1-containing PR-DUB complex, which is also a tumor suppressor and acts as a deubiquitinase for histone H2A. PR-DUB binding is compromised by cancer mutations in MLL3 and could be partially balanced by UTX in an experimental setting [11]. Recent studies have also demonstrated the interaction of UTX with the mitotic deacetylase complex (MiDAC) [12,13]. Interestingly, *UTX* escapes X inactivation in females, which is compensated for in males by the chromosome Y encoding the UTY ortholog (also referred to as KDM6C) [14,15]. Although UTY is highly similar to UTX, it only displays weak enzymatic activity [16]. Both proteins play important roles in development and disease with both the enzymatically dependent and independent functions [1,17,18]. For example, a comparison of the downstream activities of a catalytically inactive UTX mutant with the wild-type (wt) protein in a UTX-deficient BLCA cell line indicated that the tumor suppressor functions of UTX can be enzymatic-independent [19]. The human genome encodes a third human H3K27me3 demethylase JMJD3/KDM6B, which harbors a similar JmjC domain. However, JMJD3/KDM6B lacks the TPR region, and it does not incorporate into MLL3 and MLL4 complexes. In many studies, KDM6 family members play different and sometimes opposing functions in development [20,21] and in human disease [22]. Many studies have shown the prominent impact of UTX mutations on gene expression and diseases. However, whether the mRNA splicing of *UTX* could play a role in its biochemical functions has not been studied so far. Despite the identification of different mRNA isoforms in databases, a comprehensive analysis and quantitative overview of *UTX* alternative splicing events and their distribution over normal and cancerous tissues is lacking.

Here, we focused on a variety of human cell lines and tissue samples to provide the overall architecture of *UTX* mRNAs. Given the clinical significance of UTX in bladder cancer, we employed long-read sequencing of *UTX* cDNAs from different human cell lines and from normal bladder epithelium or bladder samples to define the alternative splicing region (ASR) of *UTX* mRNAs, which spans exons 12–17 [23]. We expressed the five most abundant UTX isoforms to examine their protein functions. The subcellular localization of UTX isoforms is regulated by exon 14 encoding a predicted nuclear localization sequence, or NLS. The chromatin association of certain UTX isoforms and their protein interactome is controlled by exons located in the ASR.

## 2. Materials and Methods

### 2.1. Clinical Samples, Cell Lines and Cloning

Clinical material was provided with the allowance of the RWTH centralized Biomaterial (RWTH cBMB), Medical Faculty, RWTH Aachen University. Patients’ consents were obtained before conducting the study. This study was conducted in accordance with the Declaration of Helsinki. The local ethical committee approved the protocol for this study.

The UTX long isoform cDNA was purchased from Genscript (CloneID OHu24601). The cDNA for UTX lacking exon 14 sequences was PCR amplified from pCMV-HA-UTX, which was a gift from Kristian Helin (Addgene plasmid #24168) [8]. All UTX isoforms and mutants were generated via PCR cloning into the pDONR221 vector or via site-directed mutagenesis using the QuikChange strategy (Agilent, Santa Clara, CA, USA). Inserts were transferred to the pCDNA5_FRT_TO_N-GFP destination vector using GATEWAY cloning according to the instructions of the manufacturer (Life technologies, Thermo Fisher Scientific, Waltham, MA, USA). All plasmids were validated via DNA sequence analysis, and primer sequences are available upon request.

Hela FlpIn/TRex cells were stably transfected with pOG44- and pCDNA5-based vectors for the expression of GFP-tagged UTX and were selected with blasticidin and hygromycin as described before [10,24]. RPE-1 cells were grown in DMEM: F-12 Medium (1:1); IMR-90, SH-SY5Y, MCF7 and HT-1376 in Eagle’s Minimum Essential Medium (EMEM); HEK293 and Hela in DMEM; COLO205, MV-4-11, Molm13, LNCaP and 5637 in RPMI-1640 Medium; and HCT 116 and U-2 OS in McCoy’s 5A. All media were supplemented with 10% FBS.

### 2.2. Immunofluorescence and Confocal Microscopy

Hela cells carrying GFP-tagged UTX isoforms were induced with 1 µg/mL doxycycline 24 h before the experiment. The cells were fixed in 4% formaldehyde in PBS, permeabilized with 0.1% Triton X-100 in PBS and blocked with 10% normal goat serum in PBS. DNA was stained with DAPI, and slides were imaged with ZEISS LSM880 Airyscan. ZEISS ZEN 2.3 software was used for image analysis.

### 2.3. Immunoblotting Analyses

To prepare whole-cell lysates, cells were lysed in Laemmli Buffer with 50 µg/mL DTT. Lysates were incubated at 95 °C for 10 min to denature the proteins before separation via SDS-polyacrylamide gel electrophoresis. Proteins were transferred to the nitrocellulose membrane (Invitrogen, Waltham, MA, USA) via electroblotting. The membrane was blocked in 5% skimmed milk and incubated overnight at 4 °C with primary antibodies for UTX (Cell Signaling Technologies #33510, Danvers, MA, USA), GFP (JL-8, Clontech, Danvers, MA, USA) or Vinculin (7F9, SantaCruz, CA, USA) as a loading control. Blots were incubated with corresponding secondary antibodies at concentrations suggested by the manufacturer (Bio-Rad), developed using the Clarity Western ECL kit (Bio-Rad, Hercules, CA, USA) and imaged using a ChemiDoc Touch system (Bio-Rad, Hercules, CA, USA).

### 2.4. GFP Affinity Purification and MS Sample Preparation

HeLa FlpIn/TREx cells carrying the doxycycline-inducible GFP-UTX allele were treated for 48 h with 1 µg/mL doxycycline. GFP expression was verified using immunoblotting using GFP (JL-8, Clontech) and α-tubulin (CP06, Calbiochem, San Diego, CA, USA) antibodies. Nuclear and cytoplasmic extracts were prepared for GFP-affinity purification coupled to mass spectrometry analyses as described before [8]. In brief, about 300 million cells were harvested after induction with 1 µg/mL doxycycline for 48 h, washed twice with PBS (Gibco, Thermo Fisher Scientific, Waltham, MA, USA, #10010-015), resuspended in 5 volumes of cold Buffer A (10 mM Hepes-KOH pH 7.9, 1.5 mM MgCl_2_, 10 mM KCl) and incubated for 10 min on ice. The cells were pelleted and resuspended in 2 volumes of Buffer A supplemented with 1 µM of DTT, 0.5% NP-40 and complete proteinase inhibitor (CPI, Roche, Rotkreuz, Switzerland, #11836145001, referred to as buffer A complete, hereafter). To separate nuclear and cytoplasmic lysates, the cells were then homogenized in a Dounce homogenizer on ice. The nuclear fraction was pelleted via centrifugation at 3300 g for 15 min at 4 °C. The supernatant was further cleared from debris via centrifugation at 16,000× *g* and 4 °C for 1 h and further processed as a cytoplasmic fraction. The nuclear pellet was then washed out of the cytoplasmic carryover by adding 10× volume buffer A complete and performing centrifugation at 3300× *g* for 5 min. The pellet was then resuspended and gently agitated in high salt Buffer B (420 mM NaCl, 20 mM Hepes-KOH pH 7.9, 20% *v*/*v* glycerol, 2 mM MgCl_2_, 0.2 mM EDTA, 0.1% NP40, 1 × CPI, 0.5 mM DTT) at 4 °C for 1.5 h. Subsequently, the supernatant representing the nuclear extract was obtained via centrifugation at 16,000× *g* and 4 °C for 1 h.

After Bradford protein measurement, 1 mg of the nuclear and 2 mg of the cytoplasmic fraction were used for GFP or control pulldowns, as described before [25]. GFP-coated agarose beads (Chromotek, Planegg, Germany) or control agarose beads (Chromotek) were added to the protein lysates in three replicates each and rotated overnight at 4 °C in binding buffer (20 mM Hepes-KOH pH 7.9, 300 mM NaCl, 20% glycerol, 2 mM MgCl_2_, 0.2 mM EDTA, 0.1% NP-40, 0.5 mM DTT and 1 × CPI). Thereafter, the beads were washed twice with the binding buffer containing 0.5% NP-40, twice with PBS containing 0.5% NP-40 and twice with PBS. The on-bead digestion of bound proteins was performed overnight in elution buffer (100 mM Tris-HCl pH 7.5, 2 M urea, 10 mM DTT) with 0.1 µg/mL of trypsin at RT, and eluted tryptic peptides were bound to C18 stage tips (ThermoFischer, Waltham, MA, USA) prior to mass spectrometry analysis.

### 2.5. Quantitative Mass Spectrometry Analysis

Samples were analyzed via nanoflow-LC-MS/MS on a Q-Exactive Plus coupled to an Easy-nLC 1000 or an Orbitrap Fusion Lumos coupled to an Easy-nLC 1200 nanoflow-LC-MS system (ThermoFisher Scientific, Waltham, MA, USA). A flow rate of 300 nl/min and a gradient of increasing organic proportion (buffer A: 0.1% formic acid, buffer B: 0.1% formic acid in 80% acetonitrile) in combination with a reversed-phase C18 separating column of 25 cm length was used for peptide separation. Each MS scan was followed by a maximum of 10 MS/MS scans in the data-dependent mode (TOP-10 method). Blanks were run between sample sets (e.g., between GFP and agarose control sample sets). The outcome raw files were analyzed with MaxQuant software (version 1.5.3.30). Data were aligned to the Uniprot human FASTA database [26]. Volcano plots were generated using Perseus (MQ package, version 1.5.4.0). Contaminants, reverse peptides and protein identification based on only one replication were filtered from raw data. Label-free quantification (LFQ) values were transformed to the log2 scale to generate the normal distribution of the data. Quality was checked by generating the unsupervised clustering of replicates and predicted proteins that were depicted as a heatmap for manual inspection. Scatter plots of the hits were also generated based on the Spearman’s correlation coefficient of the LFQ values to quality check the correlation between the GFP condition of each experiment. The imputation of the missing values was then performed on the normally distributed data (width = 0.3 and shift = 1.8). The significantly different proteins between GFP and agarose control pulldown proteins were calculated using a two-tailed Student’s *t*-test using 1% FDR. The constant value of 1 was kept for the threshold of significance (S0 = 1). Intensity-Based Absolute Quantification (iBAQ) values were used to calculate the stoichiometry as the subsequent relative protein abundance estimation [27]. The iBAQ values for each replication of the GFP pulldown were subtracted by the mean of the values from the agarose bead control pulldowns. The abundance of nuclear interactors was normalized based on the PAXIP1 subunit of the MLL3 and MLL4 complexes.

### 2.6. RT-PCR, Gel Electrophoresis and Nanopore Analysis of the UTX Isoforms

For cell lines, RNA was extracted using the RNeasy kit (Qiagen, Hilden, Germany), and for the clinical samples, the truXTRAC FFPE kit was used (Covaris, Woburn, MA, USA). DNase treatment was performed using the Turbo DNase kit (ThermoFisher). cDNA was generated with SuperScript III (ThermoFisher) and polydT primers. TAKARA DNA polymerase (TAKARA, Shiga, Japan) was used for PCR reactions. The entire *UTX* ORF was amplified using primers targeting 5′-UTR and 3′-UTR (Table S1) and analyzed via ethidium bromide staining after separation using a 1% agarose gel. The ASR region was amplified using primers targeting exon 12 and exon 17 of *UTX*. We note that, except for the 5637 cell line, the ASR of all other samples was only analyzed once. The observed bands were gel eluted, reamplified, cleaned up and subjected to Sanger sequencing.

The primers also included an extension of Nanopore universal tags:

5′-TTTCTGTTGGTGCTGATATTGC-[project-specific forward primer sequence]-3′

5′-ACTTGCCTGTCGCTCTATCTTC-[project-specific reverse primer sequence]-3′. For Nanopore sequencing, PCR products were further processed following the manufacturer’s instructions (PCR barcoding 96 amplicons SQK-LSK109). Next, 0.5 nM of the first-round PCR was further amplified using the Nanopore Barcoding primers (EXP-PBC096). Thereafter, the barcoded amplicons were pooled, and a 0.75× AMPure bead clean-up (A63880, Beckman Coulter) was performed to deplete unwanted fragments below 150 bp. Thereafter, the pool was subjected to NEBNext FFPE DNA Repair and Ultra II End-prep kits (M6630 and E7546, New England Biolabs, Ipswich, MA, USA). After that, Nanopore adaptors were ligated using NEBNext Quick T4 DNA ligase (E6056, New England Biolabs) and subsequently cleaned with AMPure XP beads (A63880, Beckman Coulter, Brea, CA, USA) using Nanopore’s short fragment buffer for washing the beads. The library was loaded on MinION Flow cells (FLO-MIN106, Oxford Nanopore, London, UK) using the supplied Sequencing Buffer and Loading Beads. Raw data were basecalled using Guppy (version 4.3.2). Analysis was performed using FLAIR software (version 1.5.1) (https://github.com/BrooksLabUCSC/flair, version 1.5.1, accessed on 23 June 2022). In brief, the reads were mapped to the human genome (version hg38) using minimap2 with option: -ax splice -t 30—secondary = no. Each aligned bam file was converted to a bed file using the bam2Bed12.py tool of FLAIR, and then, misaligned splice-sites were corrected with the genome annotation available at GENCODE project’s website (https://www.gencodegenes.org/, version 32, accessed on 23 June 2022). In order to identify all highly confident isoforms present in the cohort, splice-site corrected data from all samples were pooled together. After this, the collapse function of FLAIR was used to merge identical isoforms. Reads associated with these collapsed isoforms were quantified for each sample separately. Isoforms with read numbers of less than 100 or with an occurrence of <1% were filtered out.

### 2.7. Public Dataset Analysis

NLS prediction was performed using the NLS mapper tool [28]. The mutation information was collected from the cBioPortal and gnomAD web browsers [29,30,31].

### 2.8. GreenCUT&RUN

Half a million Hela-FRT cells expressing either UTX long or Δ14 isoforms as GFP fusions were harvested and washed twice in cold PBS. The cells were immobilized on concanavalin A-conjugated paramagnetic beads, permeabilized with 0.05% digitonin and subjected to the greenCUT&RUN protocol, as described before [32]. We added mononucleosomal Drosophila DNA as spike-in DNA for normalization purposes. Sequencing libraries were prepared using the NEB Next Ultra II kit (New England Biolabs, Ipswich, MA, USA). The resulting DNA quantity and size distribution was assessed using a Qubit instrument (Invitrogen, Waltham, MA, USA) and Agilent Bioanalyzer chips (DNA high sensitivity assay), respectively.

### 2.9. Bioinformatic Analyses of GreenCUT&RUN

The HeLa cell data for H3K4me3 (ID: ENCFF063XTI), H3K4me1 (ENCFF617YCQ) and H3K27ac (ENCFF113QJM) were obtained from the ENCODE consortium (https://www.encodeproject.org/, accessed on 23 June 2022) and ATAC-seq from SRA-NCBI (accession ID: SRR8171287). Quality control filtering was performed using Trim-galore (version 0.6.3) with default parameters. The good-quality reads were aligned on the human (version hg38) and Drosophila reference genome (BDFP5) using bowtie2 (version 2.3.4.1) with option: --dovetail –local --very-sensitive-local --no-unal --no-mixed --no-discordant -I 10 -X 700 [25,33]. We used HOMER for calling the narrow peaks with default parameters, except we disabled “filtering based on clonal signals” with option: -C 0. The peaks were annotated with HOMER. Those peaks present in the miRNA, ncRNA, pseudogenes, snoRNA, scRNA and rRNA were categorized as “other”. To generate heatmaps, the computeMatrix function of deeptools (version 3.3.2) was used with the default parameters, except sums of the reads were calculated per bin using option “--averageTypeBins” instead of mean. To normalize this matrix, sums of the reads were divided either by the total number of reads (if not available, e.g., ChIPseq data downloaded from ENCODE website) or by SpikeIn reads. To generate heatmaps for the ENCODE datasets, bamCompare files were used. To find the average number of reads in the background, initially, ~10,000 regions were randomly selected using shuffleBed (version 2.27.1), and overlapping reads were extracted using the getPeakTags function of HOMER. R was used to calculate the median and the median absolute deviation.

## 3. Results

### 3.1. Characterization of the UTX Alternative Splicing Events

**Figure 1:**
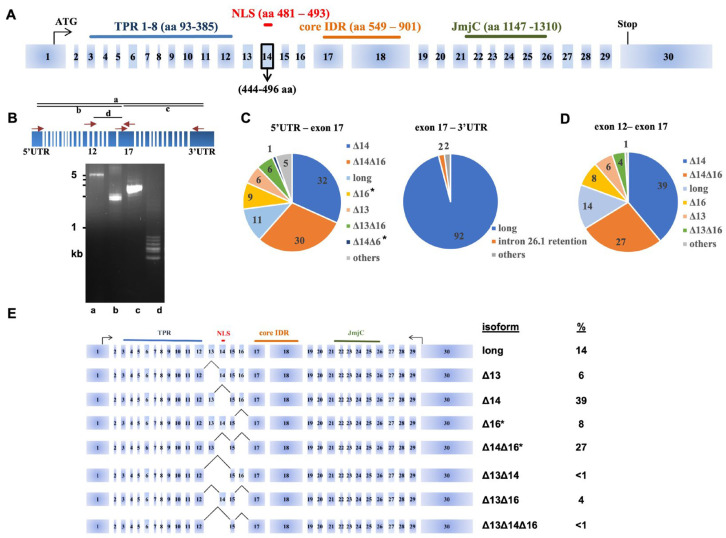
A comprehensive analysis of *UTX* alternative splicing events derived from long-read cDNA sequencing. (**A**) Exon–intron structure of the *UTX* gene with the presence of a strong NLS (indicated in red) in the commonly overlooked exon 14. (**B**) Different RT-PCR primer sets were used to amplify (parts of) the *UTX* coding region for Nanopore sequencing of RNA isolated from 5637 bladder cancer cells. Amplicon a spans the entire open reading frame of *UTX* from the 5′-UTR to the 3′-UTR, amplicon b from the 5′-UTR to exon 17, amplicon c from exon 17 to the 3′-UTR and amplicon d from exon 12 to exon 17. Arrows indicate the amplification primer location and directions. cDNA products were analyzed via ethidium bromide staining after electrophoresis on a 1% agarose gel. (**C**) Long-read sequencing results of UTX cDNAs obtained with primer sets b and c identify the alternative splicing region (ASR) spanning exons 12 through 17. The pie charts indicate the abundance in percentages of the different isoforms in 5637 cells, including two novel isoforms Δ16 and Δ14Δ16 (indicated by asterisks). ‘Others’ denote cDNAs of an abundance of 1% or less. (**D**) Pie chart of long-read sequencing results of UTX cDNAs obtained with primer set d confirmed alternative splicing events between exons 12 and 17 in 5637 cells. Numbers in the pie chart indicate the percentages. (**E**) Summary of the results based on the primer-set d are displayed on the UTX exon–intron map. Asterisks indicate the novel isoforms.

A comparison of the mRNA isoforms from the human *UTX*/*KDM6A* gene in various public databases indicated the existence of multiple alternatively spliced mRNAs. The longest isoform (NM_001291415 in the NCBI database) is used in Figure 1A to indicate all possible *UTX* exons. Exon 14, which contains a predicted NLS [28], is absent in the canonical isoform (NM_021140). Therefore, in many human UTX studies, an artificial NLS has been attached to canonical UTX to obtain robust nuclear expression, while the long isoform with the natural NLS has been overlooked [7,8,34]. A very similar pattern was observed for UTY, with a predicted NLS after the TPR at the long isoform (NM_001258249) encoded by exon 12 (nucleotides 1434-1467), which is absent from the canonical *UTY* transcript (NM_007125) [35].

To investigate the alternative splicing events and the proportion of *UTX* mRNA isoforms in a comprehensive manner, we first focused on the commonly used bladder carcinoma cell line 5637. This epithelial-like cell line has been developed from a grade II urothelial carcinoma of male origin, carrying a wild-type *UTX* allele, and contains no mutations in other members of the COMPASS family of SET1/MLL complexes or other key epigenetic genes [36]. First, we amplified the whole 4359-bp coding region of *UTX* mRNA via RT-PCR (amplicon “a”, Figure 1B and Figure S1A) and subjected the resulting cDNAs to long-read Nanopore sequencing. This experiment revealed a variety of alternative splicing events, which were confined to the central region of the *UTX* gene. The hotspot of alternative splicing occurred just after TPR and at the beginning of the middle part of *UTX* comprising exons 12 to 17. In order to increase the number of high-quality long reads, we divided the *UTX* coding region into three different regions for the RT-PCR amplification of RNAs isolated from 5637 cells. Regions b and c span exons 1 to 17 and exons 17 to 30, respectively, whereas region d covers the central region from exon 12 and exon 17 (Figure 1B). Agarose gel electrophoresis analysis already revealed distinct DNA fragments of region d cDNAs ranging from 400 to 900 bp (Figure 1B). We performed the long-read sequencing of cDNA products from regions b and c, and we calculated the percentage of different mRNA isoforms of *UTX*. A total of 86,710 reads for region b and 3898 reads for region c were obtained. Isoforms with less than 100 reads were excluded from further analysis, which was focused on cDNAs with an abundance of 1% or more. The rest were denoted as “others” in Figure 1C,D. The analysis of fragment b cDNAs identified many alternative mRNA splicing events in the region spanning exons 12–17 of the *UTX* gene, which we refer to as the alternative splicing region (ASR). The long-read sequencing of fragment c cDNAs, which span the second half of *UTX* (exons 17–30), did not reveal alternative splicing events, except the infrequent retention (2%) of intron-26-encoded sequences (Figure 1C). These findings are consistent with *UTX* transcripts annotated in the NCBI database, which did not quantify the diversity of *UTX* mRNAs in the ASR. In addition, our analysis identified two novel mRNA isoforms, Δ16 and Δ14Δ16, which together comprise more than a third of UTX mRNAs (Figure 1C). Next, we zoomed in by sequencing cDNAs (n = 93,817) from region d spanning the ASR (Figure 1D). Indeed, long-read sequencing confirmed the abundance of two novel *UTX* mRNA isoforms lacking exon 16 as well as isoform Δ14Δ16. We noted small differences in isoform percentages between amplicons of different lengths, which could have been due to a PCR bias. For proper comparisons, we focused on the smallest amplicon d for the rest of the study. To verify the long-read sequencing results, we separated individual bands of the ASR-containing fragment d via agarose gel electrophoresis and reamplified the eluted bands for confirmation via standard Sanger sequencing. This confirmed the sequences of the long Δ14, Δ16 and Δ14Δ16 isoforms (Figure S1A,B).

In conclusion, *UTX* cDNA analysis from 5637 cells showed that isoform Δ14 is the most abundant mRNA isoform, constituting 39% of the *UTX* transcripts, followed by the novel isoform Δ14Δ16 with 27%, the “long” isoform (including all 30 exons) with 14%, novel isoform Δ16 with 8%, Δ13 with 6%, Δ13Δ16 with 4% and all other isoforms at a frequency of less than 1% (Figure 1D,E).

### 3.2. Long-Read Analysis of UTX mRNAs across A Panel of Cell Lines and Bladder Samples

**Figure 2:**
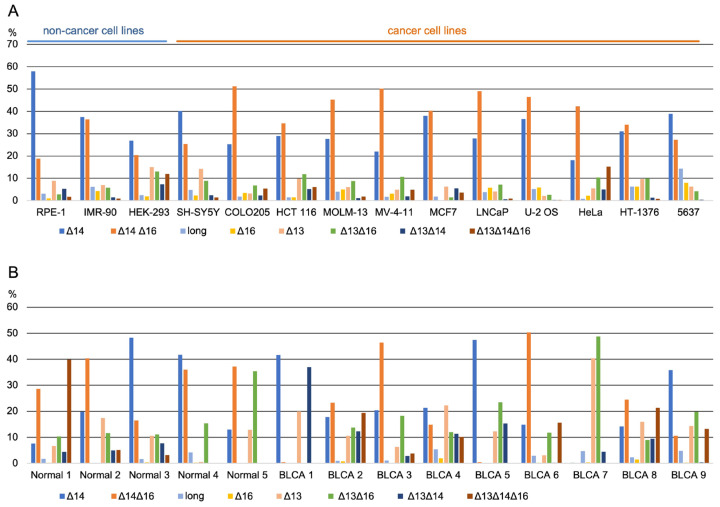
Nanopore sequencing reveals the high frequency of non-canonical isoforms in different cell lines and a high proportion of alternatively spliced transcripts at exon 14 and exon 16. (**A**) *UTX* isoform composition in different cell lines was determined via long-read sequencing of *UTX* cDNAs. The frequency of *UTX* isoforms varied between different cell lines. (**B**) *UTX* isoform composition in bladder cancer and normal bladder epithelium samples. The frequency of *UTX* isoforms demonstrates high variability in the isoform distribution in different samples.

Alternative splicing patterns can display both tissue specificity and can be altered in disease states [37]. To examine this issue for *UTX* mRNAs, we focused on the *UTX* ASR for mRNA splicing variation across a panel of human cell lines representing different tissues of origin. In total, we performed long-read sequencing on ASR (or fragment d) amplicons from 14 cell lines, 3 of which with a non-cancer origin and 11 with a cancer origin (including the bladder carcinoma cell lines 5637 and HT-1376). The read numbers per sample ranged between 11,674 for MCF7 and 125,221 for LNCaP cells (Table S2). The percentage of the longest *UTX* mRNA in 5637 and HT-1376 cells was 14% and 6% (Figure 2A), respectively, whereas the commonly used *UTX* Δ14 isoform ranged from only 18% in HeLa to 58% in the diploid retinal pigmented epithelial cells (RPE-1). The novel Δ14Δ16 *UTX* mRNA isoform ranged from 19% in RPE-1 to 51% in the colorectal carcinoma cell line, COLO205, whereas the novel Δ16 isoform was less abundant and ranged from 1% to 6% (Figure 2A).

Given the high proportion of *UTX* mutations in bladder carcinoma, we decided to investigate the distribution of *UTX* mRNA isoforms in human bladder tissues. For this, we obtained clinical samples from nine BLCAs and from five normal bladder epithelia. All BLCAs were of the muscle-invasive category, and normal epithelial samples were obtained from non-cancer individuals using laser microdissection. Nanopore sequencing of region d spanning the ASR showed high variability in different isoforms (Figure 2B). The canonical Δ14 ranged from 8 to 48% in normal bladder and 14 to 47% in BLCA, constituting an average of 26% and 24% of the isoforms in normal samples or BLCA, respectively. No significant differences were observed in the proportion of isoforms or exon percentage spliced-in (PSI) values between normal samples and tumors (Table 1). Nevertheless, our results demonstrated remarkable variability in the *UTX* mRNA isoforms across different samples. The canonical isoform Δ14, which is regarded as the predominant form of *UTX*, makes up only around a quarter of the *UTX* transcripts.

In conclusion, we identified two novel mRNA isoforms, Δ16 and Δ14Δ16, by applying the long-read sequencing of *UTX* cDNAs, which display a high level of variability in splicing isoforms between different cell lines and clinical samples. This variability in mRNA isoforms may instruct different UTX functions.

### 3.3. UTX Isoforms Display Different Protein–Protein Interactions

**Figure 3:**
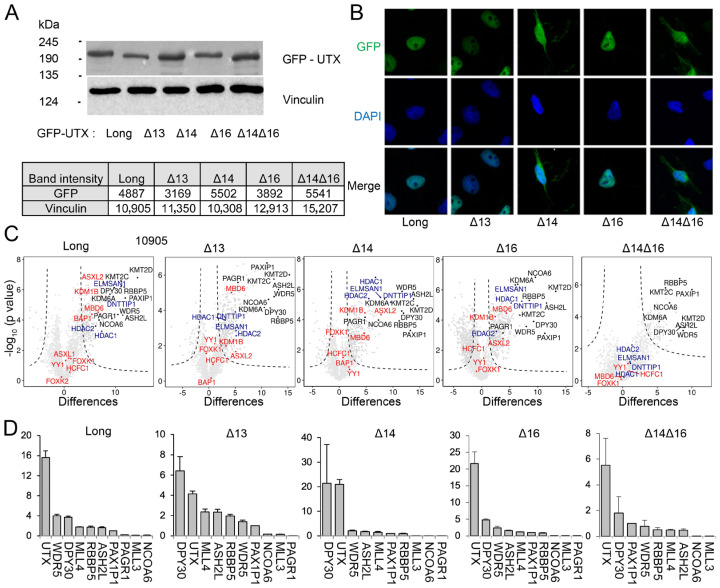
UTX isoforms have different cellular locations and protein–protein interactions. (**A**) The most abundant UTX isoforms were expressed as GFP fusions in Hela cells, and immunoblot analysis using GFP antibodies validated the expression and the sizes corresponding to the predicted molecular weights. The lower table indicates the intensity of each protein as determined via densitometry. (**B**) Fluorescence microscopy shows that UTX isoforms lacking exon 14 accumulated in the cytoplasm in contrast to isoforms containing exon 14. Micrographs were taken using the Zeiss LSM 880 microscope with objective 40×. (**C**) Protein–protein interactions of different UTX isoforms as determined via qMS. Interactions with MLL3/MLL4, PR-DUB and MiDAC complexes are marked with black, orange and purple dots, respectively. Isoform Δ14Δ16 does not interact with the MiDAC or PR-DUB complexes. (**D**) Comparison of the relative stoichiometries of the MLL3 and MLL4 subunits between UTX isoforms. Protein abundance was normalized to PAXIP1/PTIP peptides. PAGR1 was not detected in isoform Δ14Δ16. Each qMS experiment compares the mean of three GFP-bead vs. three agarose-bead control pulldowns. The error bars represent standard deviations.

To compare the protein interactome of the long isoform of UTX with isoforms lacking each of the alternative exons (13, 14 or 16) and the abundant novel UTX isoform Δ14Δ16, we expressed these UTX proteins as N-terminally GFP-tagged versions from a single chromosomal integration site in the Hela-FlpIn/T-REx cell line. The GFP-fusion format allows for both fluorescence localization and interactome experiments for UTX proteins. The immunoblotting of total lysates showed that all GFP-UTX proteins are expressed in similar levels with a slightly lower expression of isoforms Δ13 and Δ16. As expected, the long isoform showed the lowest and the Δ14Δ16 isoform displayed the highest mobility in protein gels (Figure 3A and Figure S2A). Confocal fluorescence microscopy showed a robust nuclear expression of GFP-UTX isoforms including exon 14 (Figure 3B). In contrast, isoforms lacking this exon, Δ14 and Δ14Δ16, also accumulate in the cytoplasm (Figure 3B), which suggests that the predicted NLS of exon 14 increases the nuclear localization of UTX protein. We carefully examined whether nuclear puncta, characteristic of phase separation properties of UTX, as reported before [7], could be observed in our HeLa cell system. However, we could not detect the accumulation of nuclear puncta for any of the GFP-UTX isoforms.

Next, we compared the nuclear interactomes of the five UTX isoforms by conducting GFP-affinity purifications followed by iBAQ-based quantitative mass spectrometry (qMS). Our qMS approach allowed for both the definition of significant interactors as shown by volcano plots (Figure 3C) and the quantification of the relative abundance of a significant interactor (Figure 3D) using Perseus software [38]. As expected, [10], we identified the full MLL3 and MLL4 histone H3K4 methylation complexes in the interactome of the long isoform of UTX (indicated by black dots in Figure 3C). MLL3 and MLL4 subunits also stand out as interactors with all other UTX isoforms, indicating that the sequences encoded by exons 13, 14 and 16 are not essential for MLL3/MLL4 interactions, which is confirmed by their relative stoichiometries normalized against the PAXIP1 subunit (Figure 3D). UTX seems to interact stronger with the MLL4 compared to the MLL3 complex, but this may be related to the relative expression levels of MLL3 and MLL4 in HeLa cells [10]. The NCOA6 and PAGR1 proteins are sub-stoichiometric subunits of MLL3/MLL4 complexes, as we reported before [10]. In addition, and with lower stoichiometries (Figure 3C and Figure S2B,C), we observed members of the PR-DUB H2A deubiquitinase [39] and MiDAC histone deacetylase [40] complexes as significant hits in the UTX interactomes. All four MiDAC subunits, DNTTIP, ELMSAN1, HDAC1 and HDAC2, were present in GFP-purifications of UTX long, Δ13, Δ14 and Δ16 (Figure 3C and Figure S2B). Interestingly, MiDAC subunits were completely absent from the UTX Δ14Δ16 interactome. Several but not all PR-DUB members (BAP1, MBD6, KDM1B, and ASXL2) were present in the interactomes of UTX long, Δ13, Δ14 and Δ16 (Figure 3C and Figure S2B,C). Meanwhile, the BAP1 catalytic subunit of PR-DUB was identified as a significant interactor of UTX long, it was absent with the other isoforms (Figure 3C and Figure S2C). This indicates that PR-DUB interactions are more sensitive to the absence of UTX exons 13, 14 or 16, which is in contrast to MiDAC interactions. Interestingly, both MiDAC and PR-DUB subunits were not identified with the Δ14Δ16 isoform of UTX (Figure 3C). This indicates that the combination of sequences encoded by exon 14 and 16 of UTX are involved in interactions with the PR-DUB and MiDAC histone modification complexes.

Taken together, we determined the nuclear interactors for five abundant UTX isoforms to find that they all efficiently incorporate into the MLL3 and MLL4 complexes. In addition, UTX proteins interact with members of the PR-DUB and MiDAC histone modification complexes at lower stoichiometries, and these interactions are sensitive to the loss of ASR exons.

### 3.4. The Combination of Middle Part with TPR Is Required for Proper Protein–Protein Interaction of UTX

**Figure 4:**
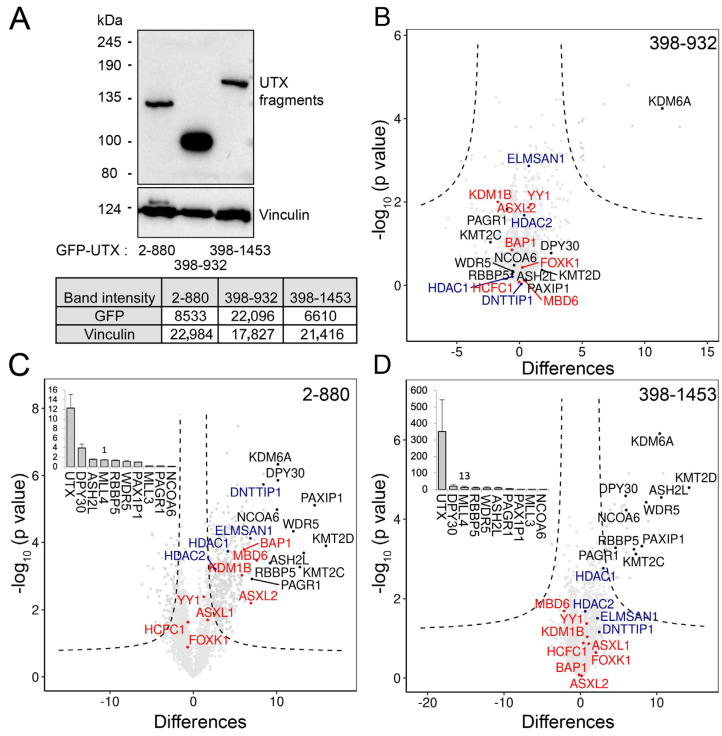
Protein interaction analysis at GFP−tagged UTX fragments. (**A**) Immunoblot analysis of GFP−tagged UTX fragments expressed in HeLa−FRT cells for qMS analysis. (**B**) UTX 398−880 (middle part) including ASR did not pull down any members of MLL3/MLL4, PR-DUB or MiDAC complexes. (**C**) UTX 2−880 (TPR and middle part) including ASR expression identified all subunits of the MLL3/MLL4, PR−DUB and MiDAC complexes. (**D**) UTX 398−1453 (middle part combined with JmjC) showed low stoichiometry interactions of the MLL3 and MLL4 complex subunits and did not interact with subunits of the PR-DUB or MiDAC complexes. Each qMS experiment compares the mean of three GFP-bead vs. three agarose-bead control pulldowns. The error bars represent standard deviation.

In order to better define the UTX regions important for the observed protein–protein interactions, we expressed GFP-tagged UTX fragments in Hela cells to perform qMS (Figure 4A and Figure S3A). First, we focused on the middle part of UTX (excluding TPR repeats and the JmjC domain and covering residues 398–932, which spans exons 12 to 18 and includes the ASR). Interestingly, the GFP fusion of this middle part did not interact with any subunit of the MLL3, MLL4, MiDAC or PR-DUB complexes (Figure 4B). When combining the TPR with the middle part (amino acids number 2–880), we observed all subunits of the above-mentioned complexes as significant interactors (Figure 4C and Figure S3B,C). In addition, the middle part plus JmjC (residues 398–1453) interacts with the MLL3 and MLL4 complexes at a low stoichiometries, when compared to the TPR with the middle part protein (Figure 4C,D). Importantly, the middle part plus JmjC of UTX does not display interactions with subunits of the PR-DUB or MiDAC complexes. Taken together, these results indicate that the middle region of UTX spanning the ASR is not able to interact independently with the subunits of the MLL3/MLL4, MiDAC or PR-DUB complexes. This region possibly stabilizes these interactions in combination with the TPR of UTX. Both the TPR and JmjC domains can mediate interactions with the MLL3 and MLL4 complexes, but the TPR seems the predominant interaction region for MiDAC and PR-DUB.

### 3.5. The UTX Long Isoform Displays A Stronger Chromatin Association When Compared to Canonical Isoform Δ14

**Figure 5:**
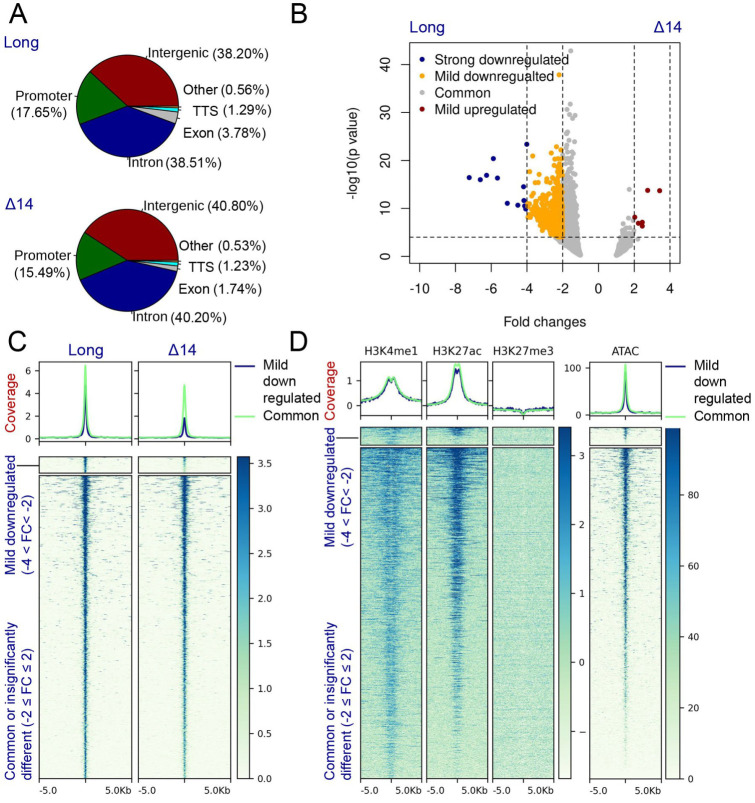
GreenCUT&RUN analysis for UTX long and Δ14 shows stronger chromatin binding via the long isoform, while both isoforms are equally enriched at the relevant histone modification sites. (**A**) Distribution of UTX binding to different genomic regions for long and Δ14 isoform. Percentages of peaks are given in brackets. (**B**) V−plot demonstrating differential peak intensity between long iso-form and Δ14 isoform. (**C**) Heatmaps showing SpikeIn normalized peak intensity of mildly down-regulated and common peaks. The number of strongly downregulated and mildly upregulated peaks are too low to be visible in heatmap. Therefore, these two categories of peaks are not shown. (**D**) Heatmaps showing coverage for H3K4me1, H3K27ac and H3K27me3 at the binding sites of differentially bind peaks. ATAC−seq signals are also shown.

In order to examine the effect of exon 14 sequences on the genome localization properties of UTX proteins, we applied greenCUT&RUN profiling [25] for UTX long and Δ14 in Hela-FRT cells (Figure 2A). The distribution of binding events over different functional genomic regions was similar between the two isoforms (Figure 5A). However, and as expected from the increased nuclear abundance, a higher number of peaks were called for UTX long compared to UTX Δ14 (n = 14,950 and n = 9070, respectively). Next, we examined whether the two isoforms display differential genomic binding by selecting regions of significant changes. For this, peaks of both isoforms were divided into five categories comparing the long isoform with the Δ14 isoform: (1) strongly downregulated (FC ≤ −4), (2) mildly downregulated (−4 < FC< −2), (3) common (−2 ≤ FC ≤ 2), (4) mildly upregulated (2 < FC < 4) and (5) strongly upregulated (FC ≥ 4). Fold changes needed to be significant (*p*-value < 0.0001); otherwise, peaks were categorized as common peaks. As shown in Figure 5B, 12 peaks were strongly downregulated and 817 peaks were mildly downregulated after the exclusion of exon 14 (Table S3). Only six peaks were mildly upregulated. This revealed that almost all peaks of the Δ14 isoform were also bound at the same intensity by the long isoform. On the other hand, peaks identified with the long isoform only were also present with the Δ14 isoform, although the intensity of peaks was different (Figure 5B,C). To determine whether any peak is specific to the long isoform, the average number of reads in the background was identified. For this, sampling was carried out, and coverage in ~10,000 random genome regions in the size range of the peaks (540 ± 100 bp) was extracted for both isoforms. On an average, we found 1 ± 1.48 (median ± median absolute deviation) reads per region. In this, when we cover 99% area of the distribution, the max value will be 1 + (3 × 1.48) ≈ 6. This suggests that 99% of the background regions will have a coverage below or equal to 6 reads. On the basis of this, we filtered peaks with a coverage of ≤6 in the Δ14 isoform but ≥12 (this value chosen to keep the fold change equal or greater than 2) in the long isoform. We only identified four peaks specific to the long isoform using these criteria. Details about these peaks are given in Table S3. Genomic tracks for the two representative UTX-long-isoform-specific sites are shown in Figure S4. The comparison of the chromatin properties of UTX-bound regions for the histone marks at the H3K4me1, H3K27ac, H3K27me3, and ATAC sites did not reveal clear differences between the UTX long and Δ14 isoforms (Figure 5D). For both mildly downregulated and common peaks, we observed robust binding to active chromatin sites, as indicated by the overlap with H3K4me1, H3K27ac and ATAC signals (Figure 5D). We note that H3K27me3 sites are under-represented at UTX sites (Figure 5D). In conclusion, the genome localization analysis indicates that exon 14 sequences do not control specific chromatin-binding properties of UTX, but rather its nuclear abundance, which is reflected by the lower peak number and intensity of UTX Δ14. Both UTX long and Δ14 isoforms bind to the active regions of the genome, as revealed by colocalization with histone H3K4me1 and H3K27ac modifications and with ATAC signals.

## 4. Discussion

Next-generation sequencing data of healthy and pathological samples, especially from cancer tissues, have indicated that *UTX*/*KDM6A* is an important gene for human diseases [1]. Molecular studies revealed that the UTX H3K27 demethylase plays a pivotal role in gene expression control as a regulator of chromatin modifications. The molecular functions of UTX are believed to be related to its interaction with the MLL3 and MLL4 complexes [11,41], which involves the TPR domain of UTX. In addition to the importance of TPR and the catalytic JmjC domains, the middle part of UTX contains an intrinsically disordered domain (IDR), which has been implicated recently in the formation of biomolecular condensates [7].

In this study, we examined the alternative splicing patterns of *UTX* mRNAs across a variety of human cell lines and bladder tissue samples. It is well known that alternative splicing provides an additional layer of complexity to the diversity of protein functions and interactions. More than 90% of human genes are subjected to alternative splicing isoforms [42]. Different protein isoforms may play different and sometimes opposing roles [43,44]. The analysis of HAVANA annotated transcripts has shown that alternative splicing affects the domain architecture in around 43% of genes [45]. The tetratricopeptide repeat superfamily was the fourth top domain to be affected by domain architecture differences between splicing isoforms.

In this study, we discovered the presence of an alternative splicing region (ASR) in *UTX* mRNAs in the middle part of the protein, which does not overlap with the core IDR (Figure 1A). Exons 13, 14 and 16 frequently alternated in *UTX* mRNAs. As a result, a variety of different *UTX* mRNA splicing patterns were observed in normal bladders, BLCA cases and different cell lines. The frequency of these isoforms was variable, and the canonical isoform lacking exon 14 comprised only a quarter of all isoforms on average. The alternative splicing of exons 14 and 16 is especially interesting, since this region is involved in protein–protein interactions of UTX with PR-DUB and MiDAC complexes (Figure 4).

For exon 14, considering its relative abundance, the presence of a predicted NLS was associated with the increased nuclear localization of the corresponding UTX isoform. Indeed, the absence of exon 14 correlates with the reduced genomic binding of UTX, while the proteins are expressed to similar levels in the cells. Since its cloning, the weak nuclear expression of an important histone modifier such as UTX has puzzled researchers [46]. To solve the issue, the ORF of *UTX* has been fused with an artificial NLS in many studies, ever since [8]. We have solved this paradox here by describing different cellular distributions for distinct UTX isoforms. Isoforms including the NLS-encoding exon 14 should be put under further focus, because it is possible that the main chromatin functions of endogenous UTX are attributed to these isoforms rather than cytoplasmic UTX. The *UTX* Δ14 isoform has been considered as the reference in different clinical studies. For this reason, there is no mutation analysis for *UTX* exon 14 in the TCGA dataset and cBioportal [47]. We found in the International Cancer Genome Consortium database that exon 14 harbors cancer mutations, such as the recurrent mutations at the chrX:45060619 locus (GRCh38, Figure S5). We also examined the cytoplasmic fraction of the GFP-UTX isoforms in qMS experiments, and we observed only weak interactions with some of the MLL3/MLL4 complex members. On the other hand, it is possible that there are specific cytoplasmic functions or there is a dynamic equilibrium between the cytoplasmic and nuclear localization of the abundant isoforms lacking exon 14, which may depend on its interaction with the MLL3 and MLL4 complexes [6].

In our study, we identified novel isoforms of *UTX* mRNAs lacking exon 16. The observation of UTX interactions with PR-DUB and MiDAC complexes is interesting, as it provides a mechanistic basis for histone modification crosstalk orchestrated by UTX. We speculate that the PR-DUB and MiDAC complexes add a regulatory layer to gene expression regulation via UTX proteins. Moreover, a different interaction spectrum of UTX isoform Δ14Δ16 was of particular importance. A special regulatory role for different UTX isoforms could be presumed which are regulated at a splicing level. The MiDAC complex has been suggested to recruit HDAC1 and HDAC2 to deacetylate H3K27ac and H4K20ac [13]. We found differences in chromatin binding between UTX long and Δ14, which may relate to differences in the nuclear abundances of these isoforms. As expected, UTX long is a nuclear-specific isoform, and most chromatin-related functions of UTX are probably attributed to this and other exon-14-including isoforms. In particular, the considerable abundance of these isoforms as revealed by our Nanopore results indicates the importance of alternative splicing for differential UTX functions in general and for overall *UTX* mRNA abundance levels in different tissues and cell types, in particular. The development of isoform-specific antibodies and cancer mutation analysis in alternative exons are among such new directions for further analysis. In addition, the expression of individual UTX isoforms would allow for the examination of differential effects on gene transcription.

Splicing factor mutation is one of the processes implicated in tumorigenesis [48]. Bladder carcinoma and uveal melanoma show the highest ratio of splice factor mutations among TCGA cancers [29]. Interestingly, *UTX* is amongst the most frequently mutated tumor suppressors in these cancer types [2]. Our study shows remarkable diversity in *UTX* mRNA isoforms when comparing samples from human cell lines and from bladder tissues. It is tempting to speculate that the observed diversity in *UTX* mRNAs is related to the overall differences in splicing factor activity in the cell types and tissue samples analyzed in our study. These observations motivate future studies linking splicing factor mutations and activity to *UTX* mRNA splicing events in cancer-susceptible tissues such as the bladder.

In conclusion, this study provides a detailed analysis of the architecture of *UTX* mRNAs in several cell types. Alternative splicing events of *UTX* pre-mRNAs are localized to exons 12 to 17, which are involved in the nuclear localization of UTX proteins and their interactions with the PR-DUB and MiDAC chromatin regulatory complexes.

## 5. Conclusions

This study shows the existence of an alternative splicing region (ASR) in the *UTX* gene. This region is located between the TPR and IDR domains. An important feature of the ASR is that a potentially strong NLS is encoded by alternative exon 14. The splicing isoform lacking exon 14 binds less strongly to chromatin, which may be related to its reduced nuclear abundance. Isoforms lacking both exon 14 and exon 16 renders the encoded protein incapable of binding the PR-DUB and MiDAC complexes. UTX isoforms differing in the ASR are expressed at different ratios in a variety of human cell lines and in samples from normal bladder epithelia and from bladder cancer cases.

## Figures and Tables

**Table 1:** Summary of Nanopore sequencing results in BLCA and normal bladder samples.

	Isoform %								% of Exon Skipping			Exon Psi		
	Δ14	Δ14Δ16	Long	Δ16	Δ13	Δ13 Δ16	Δ13 Δ14	Δ13Δ14Δ16	No Ex 13	No Ex 14	No Ex 16	Exon 13	Exon 14	Exon 16
Normal 1	8	29	2	0	7	10	4	40	61	81	79	38	19	20
Normal 2	20	40	0	0	17	12	5	5	39	70	57	60	29	42
Normal 3	48	16	2	0	11	11	8	3	33	76	31	67	24	68
Normal 4	42	36	4	0	1	15	0	0	16	78	52	82	21	47
Normal 5	13	37	0	0	13	35	0	0	49	50	73	51	49	26
mean	26	32	2	0	10	17	3	10	40	71	58	60	28	41
BLCA 1	42	0	0	0	20	0	37	0	57	79	1	42	20	99
BLCA 2	18	23	1	1	11	14	12	19	56	73	57	43	26	42
BLCA 3	20	46	1	0	6	18	3	4	31	74	69	68	26	31
BLCA 4	21	15	5	2	22	12	11	10	56	58	39	44	42	60
BLCA 5	47	1	0	0	12	23	15	0	51	63	24	48	36	75
BLCA 6	15	50	3	0	3	12	0	16	31	81	78	68	18	21
BLCA 7	0	0	5	0	40	49	5	0	94	5	50	6	94	50
BLCA 8	14	24	2	2	16	9	9	21	56	69	56	43	29	42
BLCA 9	36	11	5	0	14	20	0	13	48	60	44	52	39	55
mean	24	19	3	1	16	17	10	9	53	63	46	46	37	53

## Data Availability

The greenCUT&RUN and Nanopore datasets have been deposited to the Sequence Read Archive (SRA) portal of the NCBI with Bioproject, accession ID PRJNA952530. All mass spectrometry data were deposited to ProteomeXchange with accession number PDX041254.

## Supplementary Materials

The following supporting information can be downloaded at: https://www.mdpi.com/article/10.3390/cancers15123117/s1, Figure S1: Confirmation of the long-read sequencing results via standard Sanger sequencing; Figure S2: Stoichiometries of non-MLL3/4 subunit interactors of UTX isoforms; Figure S3: PR-DUB and DNTTIP1 complex stoichiometry of the qMS for UTX fragments; Figure S4: Representative IGV tracks; Figure S5: UTX exon 14 mutations in the ICGC and GnomAD databases for genetic mutations.; Table S1: The list of Nanopore sequencing primers; Table S2: Number of Nanopore sequencing reads for UTX transcripts; Table S3: UTX long-only binding genes (FC > 4).

## References

[1] Wang L., Shilatifard A. (2019). UTX Mutations in Human Cancer. Cancer Cell.

[2] Robertson A.G., Kim J., Al-Ahmadie H., Bellmunt J., Guo G., Cherniack A.D., Hinoue T., Laird P.W., Hoadley K.A., Akbani R. (2017). Comprehensive Molecular Characterization of Muscle-Invasive Bladder Cancer. Cell.

[3] Glaser A.P., Fantini D., Shilatifard A., Schaeffer E.M., Meeks J.J. (2017). The Evolving Genomic Landscape of Urothelial Carcinoma. Nat. Rev. Urol..

[4] Lawson A.R.J., Abascal F., Coorens T.H.H., Hooks Y., O’Neill L., Latimer C., Raine K., Sanders M.A., Warren A.Y., Mahbubani K.T.A. (2020). Extensive Heterogeneity in Somatic Mutation and Selection in the Human Bladder. Science.

[5] Li R., Du Y., Chen Z., Xu D., Lin T., Jin S., Wang G., Liu Z., Lu M., Chen X. (2020). Macroscopic Somatic Clonal Expansion in Morphologically Normal Human Urothelium. Science.

[6] Kato H., Asamitsu K., Sun W., Kitajima S., Yoshizawa-Sugata N., Okamoto T., Masai H., Poellinger L. (2020). Cancer-Derived UTX TPR Mutations G137V and D336G Impair Interaction with MLL3/4 Complexes and Affect UTX Subcellular Localization. Oncogene.

[7] Shi B., Li W., Song Y., Wang Z., Ju R., Ulman A., Hu J., Palomba F., Zhao Y., Le J.P. (2021). UTX Condensation Underlies Its Tumour-Suppressive Activity. Nature.

[8] Agger K., Cloos P.A., Christensen J., Pasini D., Rose S., Rappsilber J., Issaeva I., Canaani E., Salcini A.E., Helin K. (2007). UTX and JMJD3 Are Histone H3K27 Demethylases Involved in HOX Gene Regulation and Development. Nature.

[9] Issaeva I., Zonis Y., Rozovskaia T., Orlovsky K., Croce C.M., Nakamura T., Mazo A., Eisenbach L., Canaani E. (2007). Knockdown of ALR (MLL2) Reveals ALR Target Genes and Leads to Alterations in Cell Adhesion and Growth. Mol. Cell. Biol..

[10] Van Nuland R., Smits A.H., Pallaki P., Jansen P.W., Vermeulen M., Timmers H.T. (2013). Quantitative Dissection and Stoichiometry Determination of the Human SET1/MLL Histone Methyltransferase Complexes. Mol. Cell. Biol..

[11] Wang L., Zhao Z., Ozark P.A., Fantini D., Marshall S.A., Rendleman E.J., Cozzolino K.A., Louis N., He X., Morgan M.A. (2018). Resetting the Epigenetic Balance of Polycomb and COMPASS Function at Enhancers for Cancer Therapy. Nat. Med..

[12] Wang X., Rosikiewicz W., Sedkov Y., Mondal B., Martinez T., Kallappagoudar S., Tvardovskiy A., Bajpai R., Xu B., Pruett-Miller S.M. (2022). The MLL3/4 Complexes and MiDAC Co-Regulate H4K20ac to Control a Specific Gene Expression Program. Life Sci. Alliance.

[13] Mondal B., Jin H., Kallappagoudar S., Sedkov Y., Martinez T., Sentmanat M.F., Poet G.J., Li C., Fan Y., Pruett-Miller S.M. (2020). The Histone Deacetylase Complex MiDAC Regulates a Neurodevelopmental Gene Expression Program to Control Neurite Outgrowth. eLife.

[14] Greenfield A., Carrel L., Pennisi D., Philippe C., Quaderi N., Siggers P., Steiner K., Tam P.P., Monaco A.P., Willard H.F. (1998). The UTX Gene Escapes X Inactivation in Mice and Humans. Hum. Mol. Genet..

[15] Tricarico R., Nicolas E., Hall M.J., Golemis E.A. (2020). X- and Y-Linked Chromatin-Modifying Genes as Regulators of Sex-Specific Cancer Incidence and Prognosis. Clin. Cancer Res..

[16] Walport L.J., Hopkinson R.J., Vollmar M., Madden S.K., Gileadi C., Oppermann U., Schofield C.J., Johansson C. (2014). Human UTY(KDM6C) Is a Male-Specific Nϵ-Methyl Lysyl Demethylase. J. Biol. Chem..

[17] Shpargel K.B., Sengoku T., Yokoyama S., Magnuson T. (2012). UTX and UTY Demonstrate Histone Demethylase-Independent Function in Mouse Embryonic Development. PLoS Genet..

[18] Wang C., Lee J.E., Cho Y.W., Xiao Y., Jin Q., Liu C., Ge K. (2012). UTX Regulates Mesoderm Differentiation of Embryonic Stem Cells Independent of H3K27 Demethylase Activity. Proc. Natl. Acad. Sci. USA.

[19] Barrows D., Feng L., Carroll T.S., Allis C.D. (2020). Loss of UTX/KDM6A and the Activation of FGFR3 Converge to Regulate Differentiation Gene-Expression Programs in Bladder Cancer. Proc. Natl. Acad. Sci. USA.

[20] Yang L., Song L., Liu X., Bai L., Li G. (2018). KDM6A and KDM6B Play Contrasting Roles in Nuclear Transfer Embryos Revealed by MERVL Reporter System. EMBO Rep..

[21] Yang L., Song L.S., Liu X.F., Xia Q., Bai L.G., Gao L., Gao G.Q., Wang Y., Wei Z.Y., Bai C.L. (2016). The Maternal Effect Genes UTX and JMJD3 Play Contrasting Roles in Mus Musculus Preimplantation Embryo Development. Sci. Rep..

[22] Ntziachristos P., Tsirigos A., Welstead G.G., Trimarchi T., Bakogianni S., Xu L., Loizou E., Holmfeldt L., Strikoudis A., King B. (2014). Contrasting Roles of Histone 3 Lysine 27 Demethylases in Acute Lymphoblastic Leukaemia. Nature.

[23] Choi W., Ochoa A., McConkey D.J., Aine M., Höglund M., Kim W.Y., Real F.X., Kiltie A.E., Milsom I., Dyrskjøt L. (2017). Genetic Alterations in the Molecular Subtypes of Bladder Cancer: Illustration in the Cancer Genome Atlas Dataset. Eur. Urol..

[24] Antonova S.V., Haffke M., Corradini E., Mikuciunas M., Low T.Y., Signor L., van Es R.M., Gupta K., Scheer E., Vos H.R. (2018). Chaperonin CCT Checkpoint Function in Basal Transcription Factor TFIID Assembly. Nat. Struct. Mol. Biol..

[25] Nizamuddin S., Koidl S., Bhuiyan T., Werner T.V., Biniossek M.L., Bonvin A.M.J.J., Lassmann S., Timmers H. (2021). Integrating Quantitative Proteomics with Accurate Genome Profiling of Transcription Factors by GreenCUT&RUN. Nucleic Acids Res..

[26] Tyanova S., Temu T., Cox J. (2016). The MaxQuant computational platform for mass spectrometry-based shotgun proteomics. Nat. Protoc..

[27] Schwanhausser B., Busse D., Li N., Dittmar G., Schuchhardt J., Wolf J., Chen W., Selbach M. (2011). Global quantification of mammalian gene expression control. Nature.

[28] Kosugi S., Hasebe M., Tomita M., Yanagawa H. (2009). Systematic Identification of Cell Cycle-Dependent Yeast Nucleocytoplasmic Shuttling Proteins by Prediction of Composite Motifs. Proc. Natl. Acad. Sci. USA.

[29] Seiler M., Peng S., Agrawal A.A., Palacino J., Teng T., Zhu P., Smith P.G., Cancer Genome Atlas Research N., Buonamici S., Yu L. (2018). Somatic Mutational Landscape of Splicing Factor Genes and Their Functional Consequences across 33 Cancer Types. Cell Rep..

[30] Gao J., Aksoy B.A., Dogrusoz U., Dresdner G., Gross B., Sumer S.O., Sun Y., Jacobsen A., Sinha R., Larsson E. (2013). Integrative Analysis of Complex Cancer Genomics and Clinical Profiles Using the CBioPortal. Sci. Signal..

[31] Karczewski K.J., Francioli L.C., Tiao G., Cummings B.B., Alföldi J., Wang Q., Collins R.L., Laricchia K.M., Ganna A., Birnbaum D.P. (2020). The Mutational Constraint Spectrum Quantified from Variation in 141,456 Humans. Nature.

[32] Koidl S., Timmers H.T.M. (2021). GreenCUT&RUN: Efficient Genomic Profiling of GFP-Tagged Transcription Factors and Chromatin Regulators. Curr. Protoc..

[33] Langmead B., Salzberg S.L. (2012). Fast Gapped-Read Alignment with Bowtie 2. Nat. Methods.

[34] Lee M.G., Villa R., Trojer P., Norman J., Yan K.P., Reinberg D., Di Croce L., Shiekhattar R. (2007). Demethylation of H3K27 Regulates Polycomb Recruitment and H2A Ubiquitination. Science.

[35] Hong S., Cho Y.W., Yu L.R., Yu H., Veenstra T.D., Ge K. (2007). Identification of JmjC Domain-Containing UTX and JMJD3 as Histone H3 Lysine 27 Demethylases. Proc. Natl. Acad. Sci. USA.

[36] Nickerson M.L., Witte N., Im K.M., Turan S., Owens C., Misner K., Tsang S.X., Cai Z., Wu S., Dean M. (2017). Molecular Analysis of Urothelial Cancer Cell Lines for Modeling Tumor Biology and Drug Response. Oncogene.

[37] Baralle F.E., Giudice J. (2017). Alternative Splicing as a Regulator of Development and Tissue Identity. Nat. Rev. Mol. Cell Biol..

[38] Tyanova S., Temu T., Sinitcyn P., Carlson A., Hein M.Y., Geiger T., Mann M., Cox J. (2016). The Perseus Computational Platform for Comprehensive Analysis of (Prote)Omics Data. Nat. Methods.

[39] Scheuermann J.C., de Ayala Alonso A.G., Oktaba K., Ly-Hartig N., McGinty R.K., Fraterman S., Wilm M., Muir T.W., Muller J. (2010). Histone H2A Deubiquitinase Activity of the Polycomb Repressive Complex PR-DUB. Nature.

[40] Bantscheff M., Hopf C., Savitski M.M., Dittmann A., Grandi P., Michon A.M., Schlegl J., Abraham Y., Becher I., Bergamini G. (2011). Chemoproteomics Profiling of HDAC Inhibitors Reveals Selective Targeting of HDAC Complexes. Nat. Biotechnol..

[41] Rickels R., Wang L., Iwanaszko M., Ozark P.A., Morgan M.A., Piunti A., Khalatyan N., Soliman S.H.A., Rendleman E.J., Savas J.N. (2020). A Small UTX Stabilization Domain of Trr Is Conserved within Mammalian MLL3-4/COMPASS and Is Sufficient to Rescue Loss of Viability in Null Animals. Genes Dev..

[42] Wang E.T., Sandberg R., Luo S., Khrebtukova I., Zhang L., Mayr C., Kingsmore S.F., Schroth G.P., Burge C.B. (2008). Alternative Isoform Regulation in Human Tissue Transcriptomes. Nature.

[43] Wu S.Y., Lee C.F., Lai H.T., Yu C.T., Lee J.E., Zuo H., Tsai S.Y., Tsai M.J., Ge K., Wan Y. (2020). Opposing Functions of BRD4 Isoforms in Breast Cancer. Mol. Cell.

[44] Li J., Huang K., Hu G., Babarinde I.A., Li Y., Dong X., Chen Y.-S., Shang L., Guo W., Wang J. (2019). An Alternative CTCF Isoform Antagonizes Canonical CTCF Occupancy and Changes Chromatin Architecture to Promote Apoptosis. Nat. Commun..

[45] Light S., Elofsson A. (2013). The Impact of Splicing on Protein Domain Architecture. Curr. Opin. Struct. Biol..

[46] Wiedemuth R., Thieme S., Navratiel K., Dorschner B., Brenner S. (2018). UTX-Moonlighting in the Cytoplasm?. Int. J. Biochem. Cell Biol..

[47] Cerami E., Gao J., Dogrusoz U., Gross B.E., Sumer S.O., Aksoy B.A., Jacobsen A., Byrne C.J., Heuer M.L., Larsson E. (2012). The CBio Cancer Genomics Portal: An Open Platform for Exploring Multidimensional Cancer Genomics Data. Cancer Discov..

[48] Ryan M., Wong W.C., Brown R., Akbani R., Su X., Broom B., Melott J., Weinstein J. (2016). TCGASpliceSeq a Compendium of Alternative MRNA Splicing in Cancer. Nucleic Acids Res..

