# Supplementary material for: Alternative mRNA Splicing Controls the Functions of the Histone H3K27 Demethylase UTX/KDM6A
Source: Cancers (Basel). 2023 Jun 8;15(12):3117. doi: 10.3390/cancers15123117 (PMC10296593; doi:10.3390/cancers15123117)
Supplement: Supplementary file 1 [file cancers-15-03117-s001.zip › Supp Figures - revised.pptx]

## Slide 1
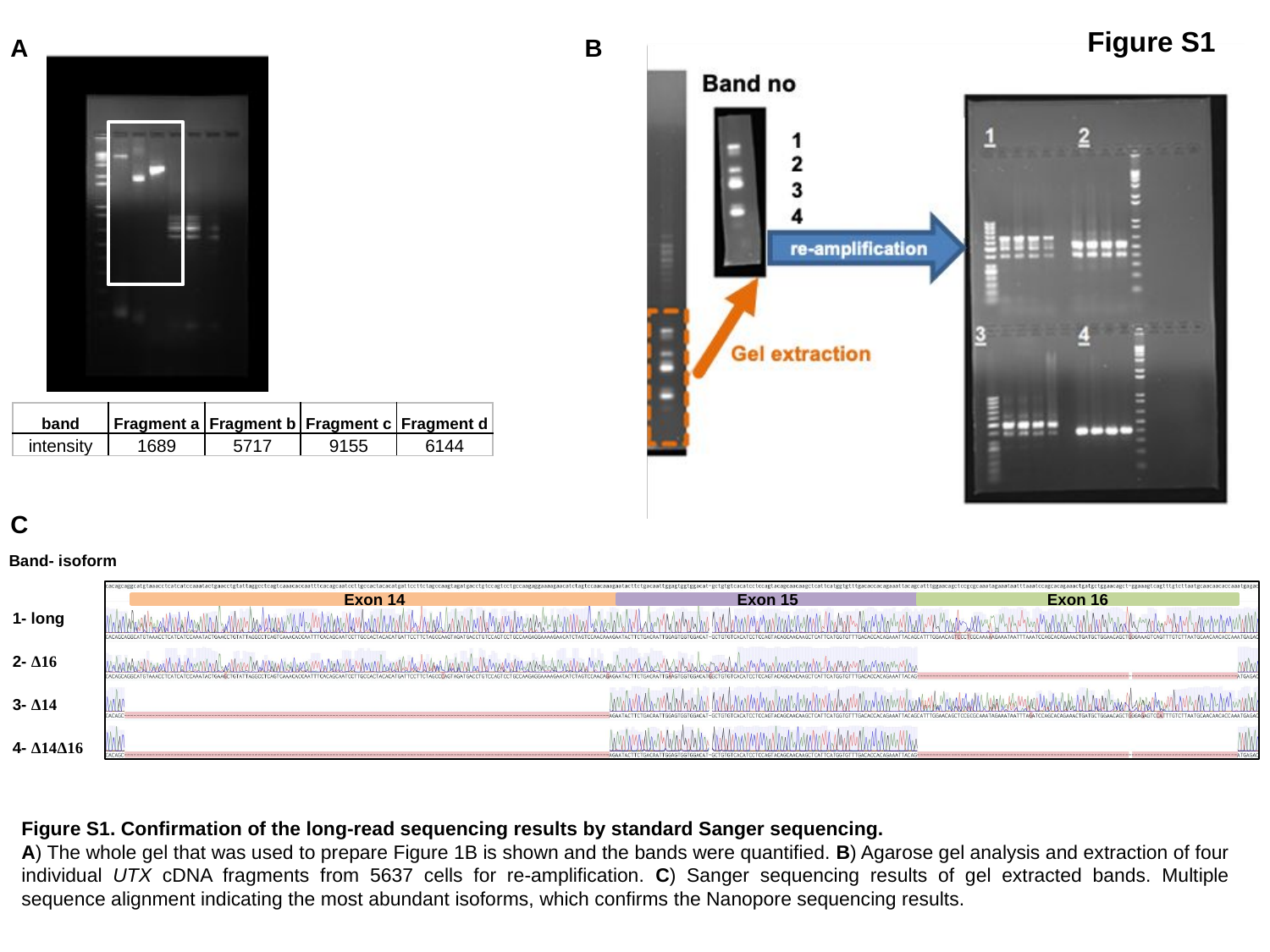

Figure S1
B
A
| band | Fragment a | Fragment b | Fragment c | Fragment d |
| --- | --- | --- | --- | --- |
| intensity | 1689 | 5717 | 9155 | 6144 |
C
Band- isoform
Exon 15
Exon 16
Exon 14
1- long
2- Δ16
3- Δ14
4- Δ14Δ16
Figure S1. Confirmation of the long-read sequencing results by standard Sanger sequencing.
A) The whole gel that was used to prepare Figure 1B is shown and the bands were quantified. B) Agarose gel analysis and extraction of four individual UTX cDNA fragments from 5637 cells for re-amplification. C) Sanger sequencing results of gel extracted bands. Multiple sequence alignment indicating the most abundant isoforms, which confirms the Nanopore sequencing results.

## Slide 2
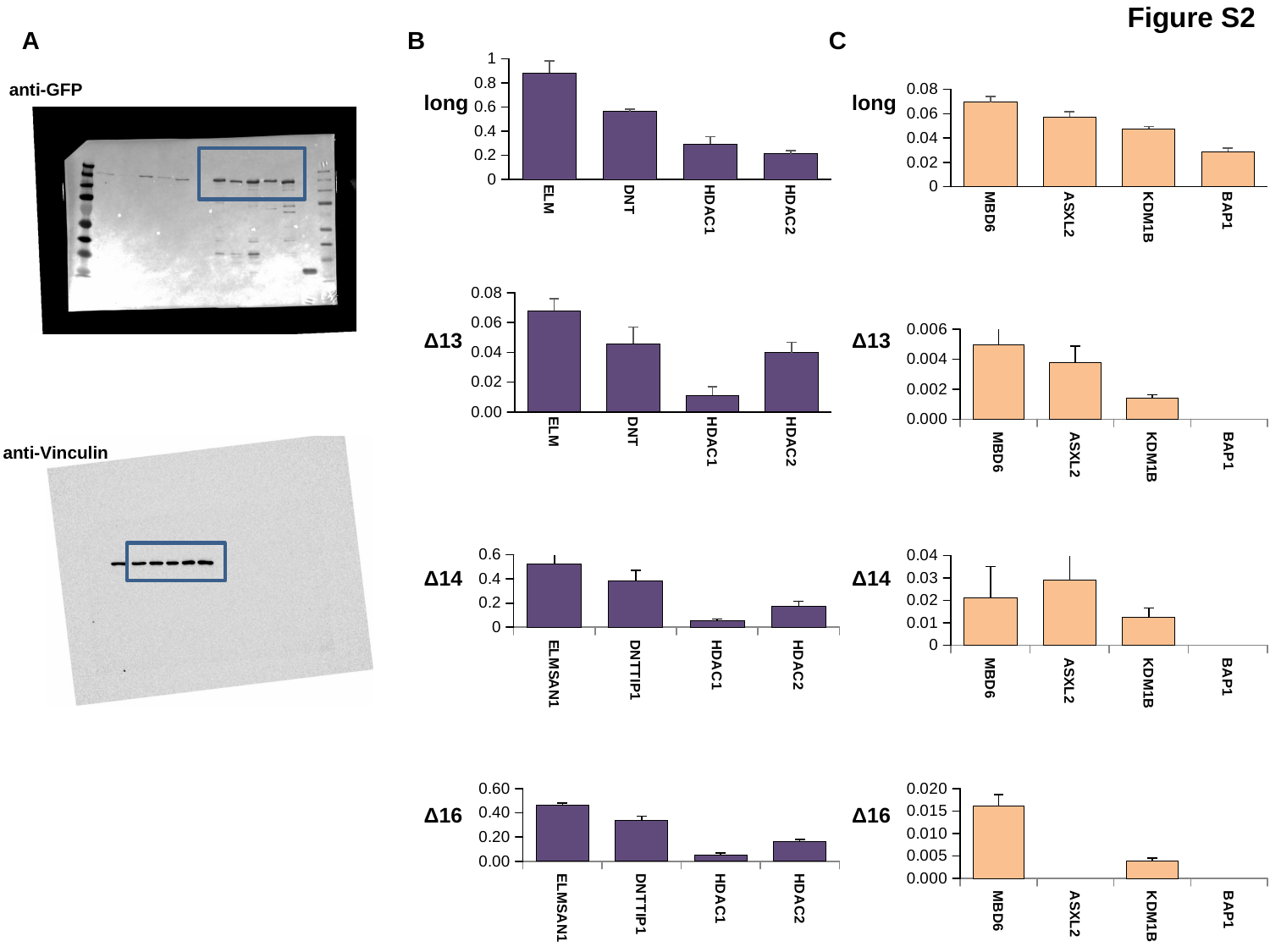

Figure S2
A
B
C
### Chart: long
| Category | |
|---|---|
| ELMSAN1 | 0.8817156491079414 |
| DNTTIP1 | 0.5620482467210265 |
| HDAC1 | 0.2929597095895046 |
| HDAC2 | 0.21467170816178002 |
### Chart: long
| Category | |
|---|---|
| MBD6 | 0.06946329307649197 |
| ASXL2 | 0.057313615750616144 |
| KDM1B | 0.047724482398196814 |
| BAP1 | 0.028729921679834413 |anti-GFP
long
long
### Chart: long
| Category | |
|---|---|
| ELMSAN1 | 0.06778527434952368 |
| DNTTIP1 | 0.0457443258917924 |
| HDAC1 | 0.011094810267381619 |
| HDAC2 | 0.03969907561634488 |
### Chart: Δ13
| Category | |
|---|---|
| MBD6 | 0.004971167216826672 |
| ASXL2 | 0.003775743364469191 |
| KDM1B | 0.0013827901810660043 |
| BAP1 | None |Δ13
Δ13
anti-Vinculin
### Chart: Δ14
| Category | |
|---|---|
| ELMSAN1 | 0.5239810524193352 |
| DNTTIP1 | 0.3799745876430962 |
| HDAC1 | 0.05628144483305995 |
| HDAC2 | 0.1724385042884041 |
### Chart: Δ14
| Category | |
|---|---|
| MBD6 | 0.02114565464062455 |
| ASXL2 | 0.028932026050289616 |
| KDM1B | 0.012312467096852906 |
| BAP1 | None |Δ14
Δ14
### Chart: Δ16
| Category | |
|---|---|
| ELMSAN1 | 0.46622574147286405 |
| DNTTIP1 | 0.33747361038351165 |
| HDAC1 | 0.051458375851081865 |
| HDAC2 | 0.16587198570214026 |
### Chart: Δ16
| Category | |
|---|---|
| MBD6 | 0.016074201809291633 |
| ASXL2 | None |
| KDM1B | 0.0037952353938946926 |
| BAP1 | None |Δ16
Δ16

## Slide 3
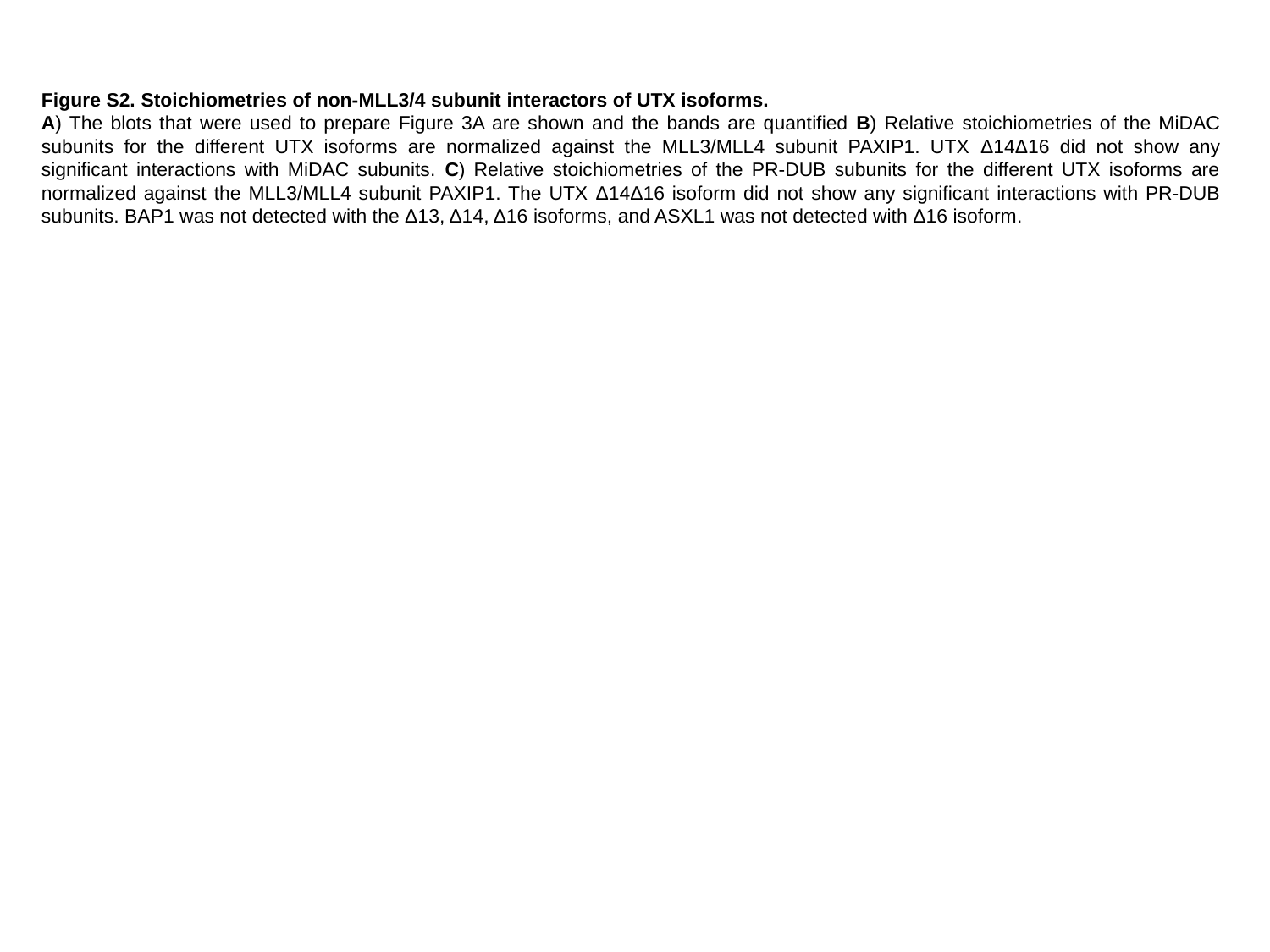

Figure S2. Stoichiometries of non-MLL3/4 subunit interactors of UTX isoforms.
A) The blots that were used to prepare Figure 3A are shown and the bands are quantified B) Relative stoichiometries of the MiDAC subunits for the different UTX isoforms are normalized against the MLL3/MLL4 subunit PAXIP1. UTX Δ14Δ16 did not show any significant interactions with MiDAC subunits. C) Relative stoichiometries of the PR-DUB subunits for the different UTX isoforms are normalized against the MLL3/MLL4 subunit PAXIP1. The UTX Δ14Δ16 isoform did not show any significant interactions with PR-DUB subunits. BAP1 was not detected with the Δ13, Δ14, Δ16 isoforms, and ASXL1 was not detected with Δ16 isoform.

## Slide 4
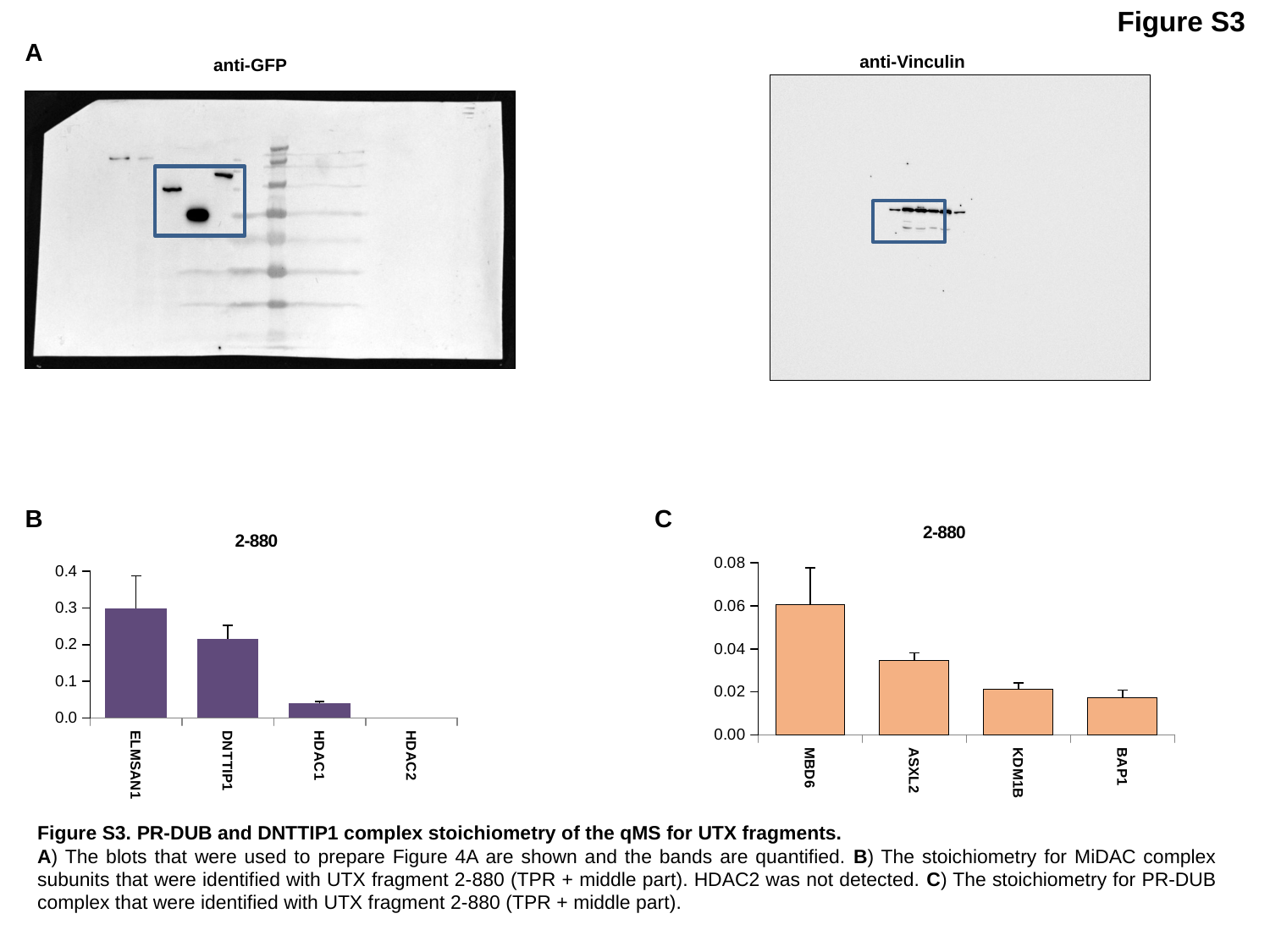

Figure S3
A
anti-Vinculin
anti-GFP
B
C
### Chart: 2-880
| Category | |
|---|---|
| MBD6 | 0.06069456222678057 |
| ASXL2 | 0.03463254632811998 |
| KDM1B | 0.021217791929193056 |
| BAP1 | 0.01719778001989704 |
### Chart: 2-880
| Category | |
|---|---|
| ELMSAN1 | 0.2984354658253587 |
| DNTTIP1 | 0.21603853617814076 |
| HDAC1 | 0.03994523047332508 |
| HDAC2 | None |Figure S3. PR-DUB and DNTTIP1 complex stoichiometry of the qMS for UTX fragments.
A) The blots that were used to prepare Figure 4A are shown and the bands are quantified. B) The stoichiometry for MiDAC complex subunits that were identified with UTX fragment 2-880 (TPR + middle part). HDAC2 was not detected. C) The stoichiometry for PR-DUB complex that were identified with UTX fragment 2-880 (TPR + middle part).

## Slide 5
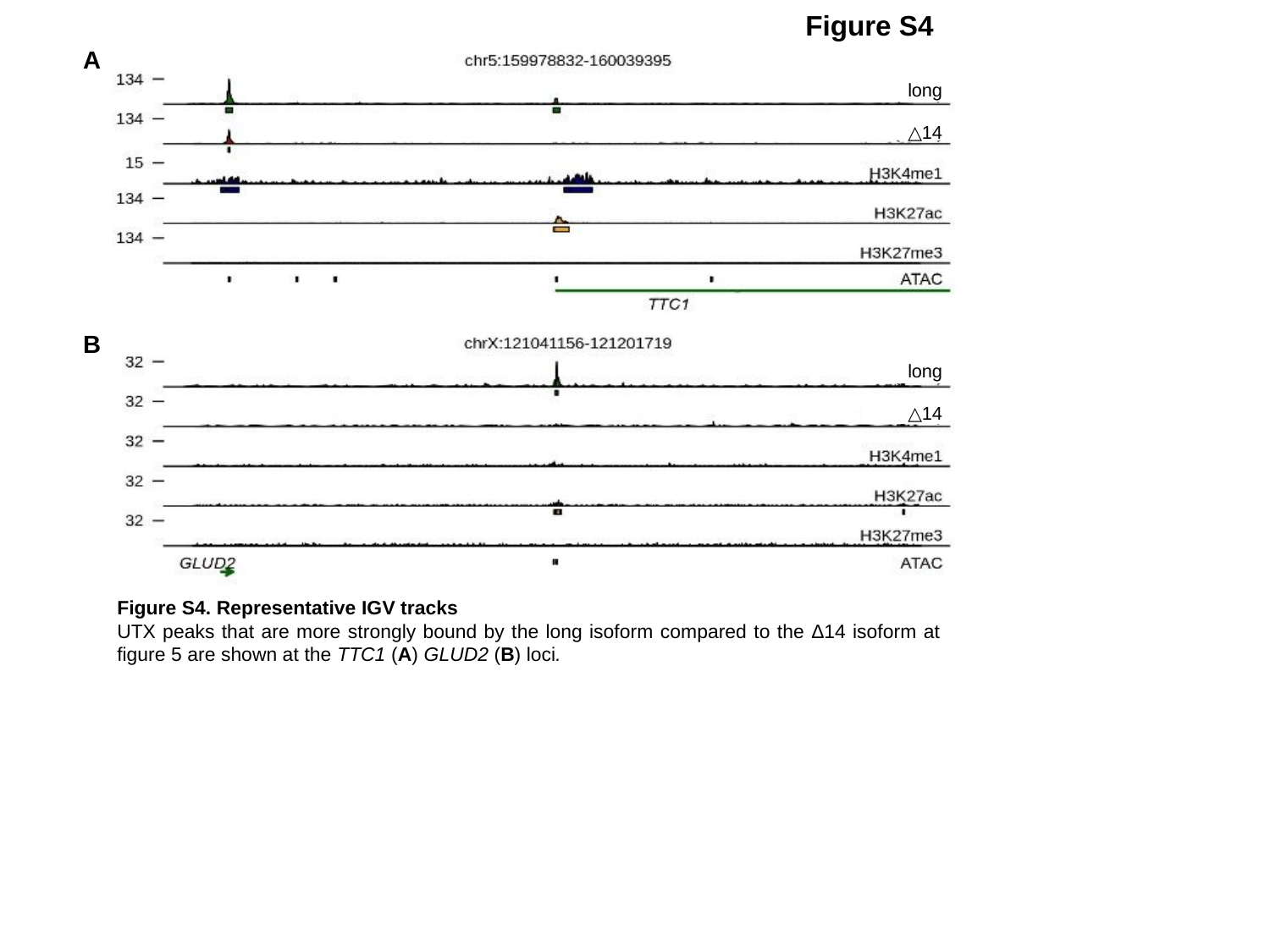

Figure S4
A
long
△14
B
long
△14
Figure S4. Representative IGV tracks
UTX peaks that are more strongly bound by the long isoform compared to the Δ14 isoform at figure 5 are shown at the TTC1 (A) GLUD2 (B) loci.

## Slide 6
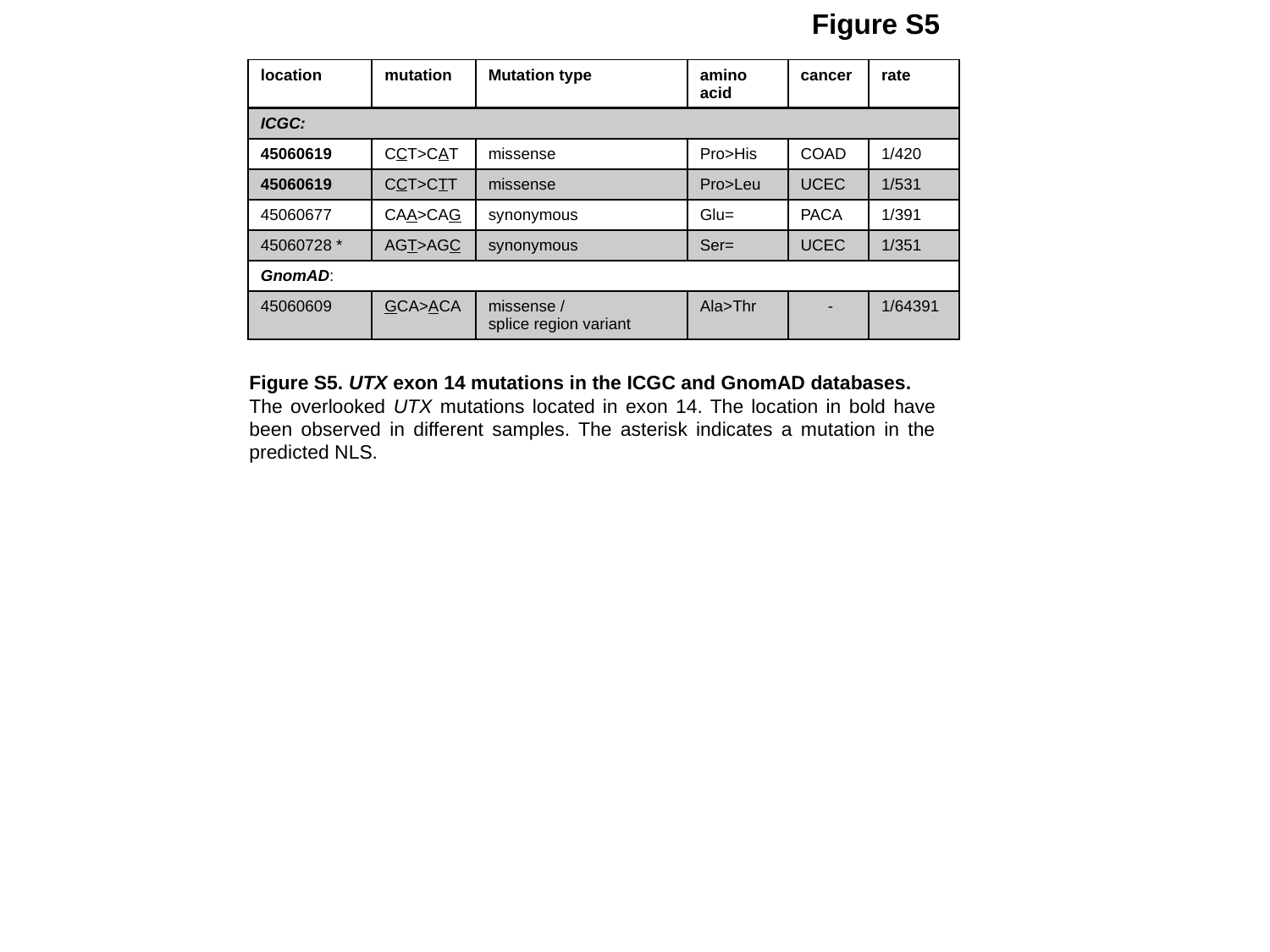

Figure S5
| location | mutation | Mutation type | amino acid | cancer | rate |
| --- | --- | --- | --- | --- | --- |
| ICGC: | | | | | |
| 45060619 | CCT>CAT | missense | Pro>His | COAD | 1/420 |
| 45060619 | CCT>CTT | missense | Pro>Leu | UCEC | 1/531 |
| 45060677 | CAA>CAG | synonymous | Glu= | PACA | 1/391 |
| 45060728 \* | AGT>AGC | synonymous | Ser= | UCEC | 1/351 |
| GnomAD: | | | | | |
| 45060609 | GCA>ACA | missense / splice region variant | Ala>Thr | - | 1/64391 |
Figure S5. UTX exon 14 mutations in the ICGC and GnomAD databases.
The overlooked UTX mutations located in exon 14. The location in bold have been observed in different samples. The asterisk indicates a mutation in the predicted NLS.
